# Prognostic value of late gadolinium enhancement cardiac magnetic resonance in hypertrophic cardiomyopathy: a meta-analysis

**DOI:** 10.1186/1532-429X-18-S1-P117

**Published:** 2016-01-27

**Authors:** Zhen Weng, Raymond H Chan, Jialu Yao, Yafeng Zhou, Yang He

**Affiliations:** 1 Cyrus Tang Hematology Center and Ministry of Education Engineering Center of Hematological Disease, Suzhou, China; 2 Cardiology, Toronto General Hospital, Toronto, ON Canada; 3 Cardiology, First Affiliated Hospital of Soochow University, Soochow, China

## Background

Contrast-enhanced cardiovascular magnetic resonance (CMR) with late gadolinium enhancement (LGE) has emerged as an *in vivo* marker of myocardial fibrosis, although its significance in identifying high risk hypertrophic cardiomyopathy (HCM) patients remains unresolved. Previous meta-analyses have included studies with data involving overlapping patient populations, thus confounding effect estimates.

## Methods

We searched PubMed and Web of Science for clinical trials that investigated the prognostic utility of LGE in HCM patients. We excluded studies with overlapping data. Pooled odds ratios (ORs), hazard ratios (HRs) and 95% confidence intervals (CIs) were calculated to assess the role of LGE CMR in the risk stratification of HCM patients.

## Results

Five studies of unique cohorts were retrieved from 393 citations for the analysis. In total, 2993 patients (mean age = 54.6 years; median follow up = 36.8 months) were included. After synthesizing data, meta-analysis showed that the presence of LGE was associated with a significantly increased risk of sudden cardiac death (SCD)/aborted SCD (pooled OR = 3.42, 95%CI = 1.97-5.94; P < 0.001), cardiac death (pooled OR = 2.93, 95%CI = 1.53-5.61; P = 0.001), all-cause mortality (pooled OR=1.80, 95%CI = 1.21-2.69; P = 0.004), and a trend towards increased risk of heart failure death (pooled OR = 2.21, 95%CI = 0.84-5.80; P = 0.107). Three publications reported results with quantitative LGE. There was a significant relationship between the extent of LGE and risk of SCD (pooled HR 1.56/10% LGE, 95% CI = 1.33-1.82, p < 0.0001), all-cause mortality (pooled HR 1.29/10%LGE, 95% CI = 1.09-1.51, p = 0.002), heart failure mortality (pooled HR 1.61/10% LGE, 95% CI 1.21-2.13, p = 0.001), and cardiovascular mortality (pooled HR 1.57/10% LGE, 95% CI 1.30-1.89, p < 0.001). After adjusting for baseline characteristics, the extent of LGE remained a strong independent predictor for SCD events (pooled HR_adjusted_ 1.36/10%LGE, 95% CI 1.10-1.69, p = 0.005).

## Conclusions

Extensive LGE by CMR identifies high-risk HCM patients, and is an independent predictor of sudden death. Quantitative assessment of myocardial fibrosis by LGE can thus be a clinically useful tool to help risk stratify patients with HCM.

**Figure 1:**
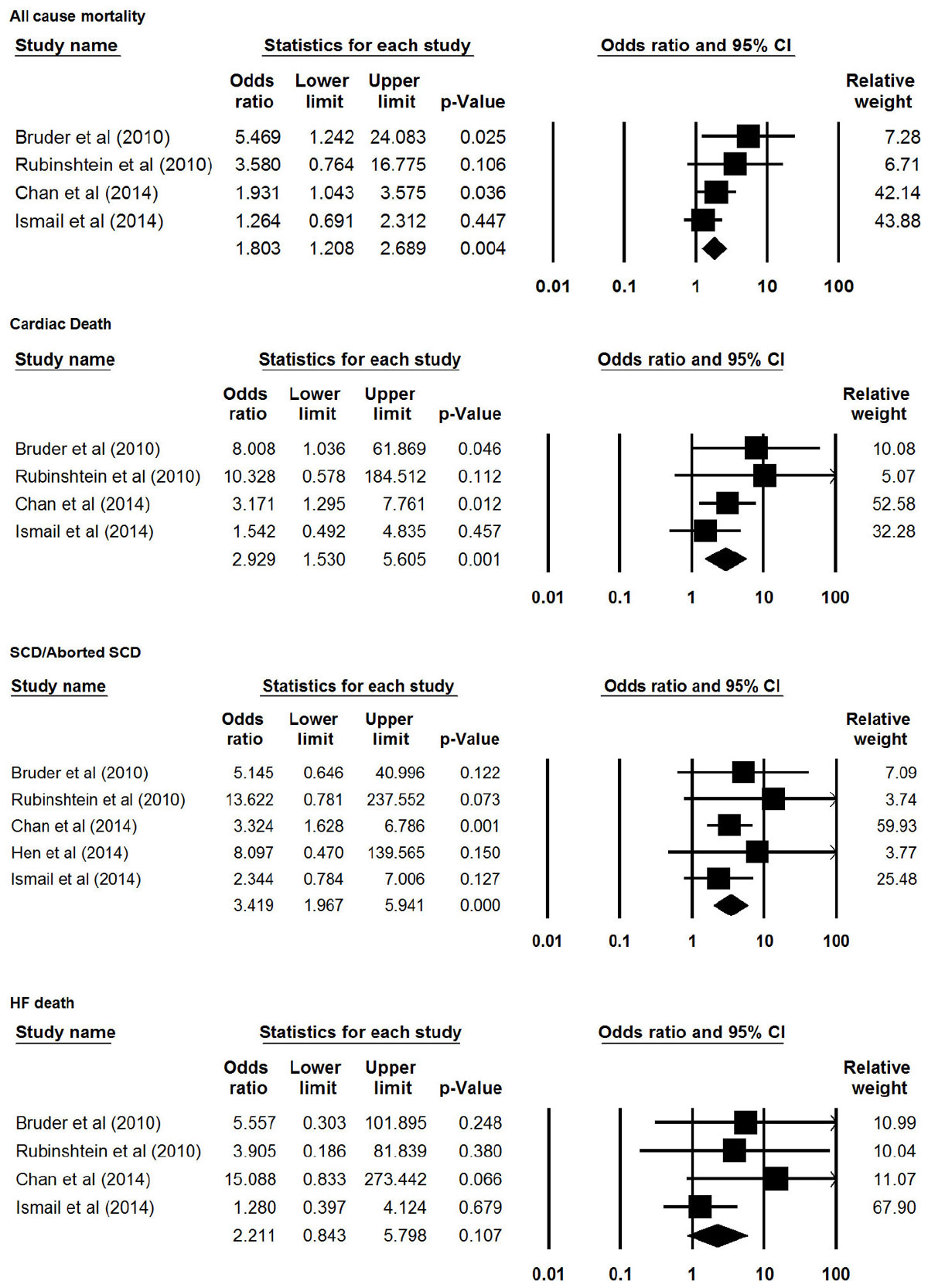
**Forest plot of presence of LGE and risk of adverse events**.

